# Qualitative and Quantitative Analysis of Regional Cerebral Free Fatty Acids in Rats Using the Stable Isotope Labeling Liquid Chromatography–Mass Spectrometry Method

**DOI:** 10.3390/molecules25215163

**Published:** 2020-11-06

**Authors:** Ting Hu, Quanfei Zhu, Yuning Hu, Ghulam Mustafa Kamal, Yuqi Feng, Anne Manyande, Jie Wang, Fuqiang Xu

**Affiliations:** 1 State Key Laboratory of Magnetic Resonance and Atomic and Molecular Physics, National Center for Magnetic Resonance in Wuhan, Key Laboratory of Magnetic Resonance in Biological Systems, Wuhan Institute of Physics and Mathematics, Innovation Academy for Precision Measurement Science and Technology, Chinese Academy of Sciences, Wuhan 430071, China; huting115@mails.ucas.ac.cn; 2 University of Chinese Academy of Sciences, Beijing 100049, China; 3 Department of Chemistry, Wuhan University, Wuhan 430072, China; qfzhu@must.edu.mo (Q.Z.); yuninghu@whu.edu.cn (Y.H.); yqfeng@whu.edu.cn (Y.F.); 4 Department of Chemistry, Khwaja Fareed University of Engineering and Information Technology, Rahim Yar Khan 64200, Pakistan; mustafa.kamal@kfueit.edu.pk; 5 Frontier Science Center for Immunology and Metabolism, Wuhan University, Wuhan 430072, China; 6 School of Human and Social Sciences, University of West London, Middlesex TW89GA, UK; Anne.Manyande2@uwl.ac.uk; 7 Center for Excellence in Brain Science and Intelligence Technology, Chinese Academy of Sciences, Shanghai 200031, China

**Keywords:** fatty acids, brain, liquid chromatography–mass spectrometry method, stable isotope labeling, regions

## Abstract

Free fatty acids serve as important bioactive molecules in the brain. They are involved in message transfer in the brain. There are many reports available in the literature regarding the role of cerebral fatty acids in message transfer; however, most of the studies are mainly focused on limited fatty acid species or only a few specific brain regions. To understand the relationship between cerebral functions and free fatty acids, it is necessary to investigate the distribution of the free fatty acids among different regions in the whole brain. In this study, free fatty acids were extracted from different brain regions and analyzed qualitatively and quantitatively using the stable isotopic labeling liquid chromatography–mass spectrometry approach. In total, 1008 potential free fatty acids were detected in the whole brain out of which 38 were found to be commonly present in all brain regions. Among different brain regions, the highest and the smallest amounts of potential free fatty acids were detected in the olfactory bulb and cerebellum, respectively. From a statistical point of view, 4-methyl-2-oxovaleric acid, *cis*-11, 14-eicosadienoic acid, tridecanoic acid, myristic acid, nonadecanoic acid, and arachidic acid were found to significantly vary among the four different brain regions (olfactory bulb, occipital lobe, hippocampus, and cerebellum). The variation in the composition of free fatty acids among different brain regions may be very important for investigating the relationship between free fatty acids and functions of cerebral regions.

## 1. Introduction

The brain is a remarkable organ that controls all functions of the body. Based on different functions, the whole brain can be divided into different regions: the olfactory bulb, which deals with smelling signals [1,2]; the occipital lobe, which is linked with the visual perception center [3,4]; the hippocampus, which is involved in learning and memory [5]; the cerebellum, which controls movement [6,7,8], and so on. Thus, it is obvious that the main function of each brain region varies significantly. This is because characteristics of cell types of different regions are different from one another. Similarly, metabolites and signaling molecules of different regions are characteristics of that specific region.

The diversity in cerebral functions depends on differences in the biochemical composition of each brain region. Lipids constitute about 50% of the brain’s dry weight and are involved in the brain structure, signal transmission, and many other functions. Fatty acids belong to lipids and can be classified in terms of either the length of their carbon chain or the degree of saturation. Based on their carbon chain length, fatty acids can be classified into a very long chain (>22 C), long chain (13–22 C), medium chain (6–12 C), and short chain (2–5 C). Whereas in terms of the degree of saturation, we can classify them into saturated (no C–C double bonds), monounsaturated (1 C–C double bonds), and polyunsaturated (>1 C–C double bonds). Polyunsaturated fatty acids evidently play an important role in the brain [9]. For example, docosahexaenoic acid (DHA) is associated with cognition [10,11,12] and arachidonic acid (AA) is involved in brain development [9]. AA can esterify cholesterol [13,14] and metabolizes into some eicosanoids, which are involved in the inflammatory response [15,16]. Protectin D1, a metabolite of DHA, is an anti-inflammatory agent [17]. Free fatty acids (FFAs) also affect signal transmission as second messengers [18,19]. A metabolite of AA, 12-Hydroxyeicosatetraenoic acid, is a neuromodulator that can protect neurons from excitotoxicity by activating a Gi/o-protein-coupled receptor [20]. Thus, qualitative and quantitative analysis of regional cerebral free fatty acids is very important for investigating cerebral functions. Furthermore, free fatty acids can also make up more complex lipids, such as phospholipids, which make up the cytoskeleton. Progression of many neurological diseases is accompanied by phospholipid abnormalities [21,22,23,24]. Phospholipids can release fatty acids by the phospholipase activity on the cell membrane [25].

Many reports are available in the literature that show the contents of FFAs in the brain. However, only a few have focused on types of FFAs or brain regions [26,27]. The diversity and distribution of free fatty acids in all brain regions have not yet been fully explored. It is, therefore, necessary to analyze the FFAs of all brain regions in order to understand the functions of various regions. For exploring the various brain functions, a variety of fatty acids are required; nevertheless, the brain tissues containing these fatty acids are limited. The mass of different brain regions varies. For example, the olfactory bulb of an adult rat weighs less than 100 mg, which is not easy to examine. In order to study the maximum possible types of fatty acids, a highly sensitive method is needed. Chemical isotope labeling liquid chromatography–mass spectrometry (CIL-LC-MS) is a suitable technique for this [28]. Utilizing this method, prior to LC-MS analysis, a pair of isotope-coded reagents is used to react with a specific functional group to form derivatives. The derivatives enhance the ionization efficiency in an Electrospray Ionizer (ESI) due to the addition of an easily ionizable group onto the target compounds. This also improves selectivity by introducing an isotope tag onto the structure. This method has been applied in various samples for less abundant targets.

To explore how free fatty acids are distributed in different brain regions, the rat brain was divided into 12 different brain regions and the medulla oblongata was selected as the research material. Ultimately, all regions were studied using qualitative and quantitative methods of FFAs for processing CIL-LC-MS data. In the whole brain, a total of 1008 potential FFAs were detected. The classes of FFAs can vary from region to region. The variation in the contents of FFAs in four different brain regions were further investigated. Six positively identified FFAs were statistically significantly different. The regional specificity of FFAs can be linked to differences in brain functions. This study could reveal a more comprehensive understanding of the distribution of FFAs in brain regions, thereby providing a reference for subsequent studies.

## 2. Results and Discussion

### 2.1. Screening and Identification of FFAs in Different Brain Regions

**Figure 1:**
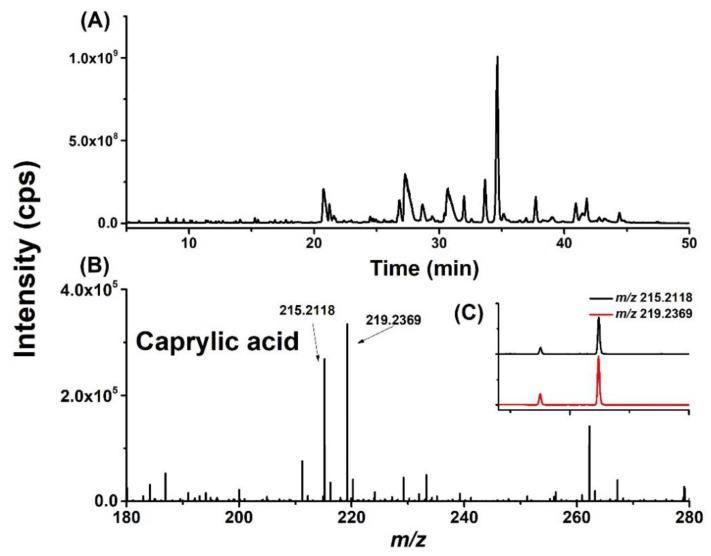
An example for the steps of identification of metabolites in brain extracts with the chemical isotope-labeled method using LC-MS under the full scan mode. (**A**) Total ion chromatograms (TIC) of the DMED/*d*_4_-DMED-labeled brain sample. (**B**) Mass spectra of DMED/*d*_4_-DMED-labeled caprylic acid. (**C**) Extracted ion chromatograms (EIC) of caprylic acid at *m*/*z* 215.2118 and 219.2369 from DMED/*d*_4_-DMED-labeled brain sample.

FFAs were firstly screened and qualitatively identified in all regions by the CIL-LC-MS method, which has been described in detail in our previous work [28]. The supernatant of 100 mg tissue from each region was equally divided into two parts and the two parts were labeled with DMED or *d*_4_-DMED, respectively. The labeled samples were mixed together before LC-MS analysis. The total ion chromatograms of the full scan are shown in Figure 1A. A potential fatty acid was detected as paired-peaks with the same retention index, similar intensities, and defined mass difference of 4.025. For example, the extracted ion chromatograms at *m*/*z* 215.2118/219.2369 have the same RIs and similar peak intensities (Figure 1C), which are considered as a potential fatty acid. In addition, precursor ions of the peak pairs were further confirmed by MS spectra of DMED/*d*_4_-DMED labeled samples (Figure 1B).

**Figure 2:**
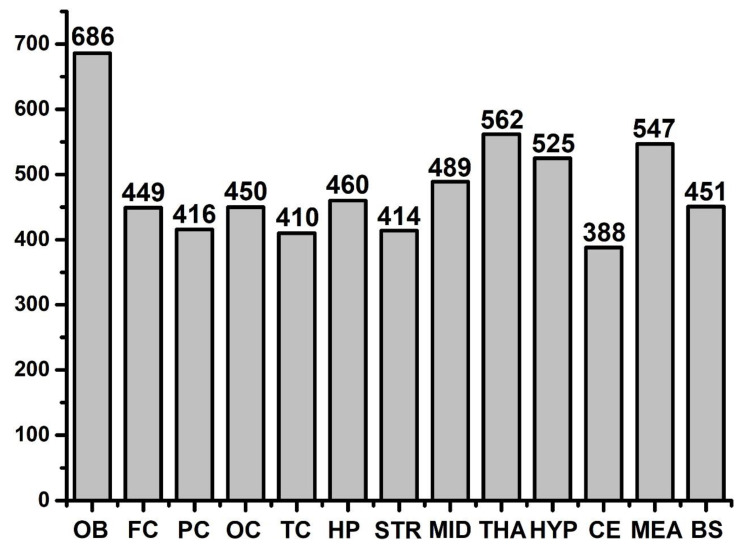
Total species of free fatty acids among different brain regions. Note: OB—olfactory bulb; FC—frontal cortex; PC—parietal cortex; OC—occipital cortex; TC—temporal cortex; HP—hippocampus; STR—striatum; MID—midbrain; THA—thalamus; HYP—hypothalamus; CE—cerebellum; MEA—medulla-pons; BS—brainstem. OB has the largest types of fatty acids and CE has the least types of fatty acids.

Finally, a total of 1008 potential FFAs were found in the whole brain. Detailed information is provided in Table S1. Among different brain regions, the following number of FFAs were detected: 686 in the OB, 449 in FC, 416 in PC, 450 in OC, 410 in TC, 414 in STR, 562 in THA, 525 in HYP, 489 in MID, 388 in CE, 460 in HP, 451 in BS, and 547 in the medulla. Furthermore, the number of FFAs of all brain regions are summarized in Figure 2. The OB has more free fatty acids than all the other regions and is an important part of the olfactory system especially for rodents. Rodents’ feeding and reproduction are dependent on the olfactory bulb. There are various cell subtypes to handle the variety of information received from the environment. It could be hypothesized that this large amount and various types of FFAs are probably needed to form various molecules to fulfil the higher demand on the OB as compared to the other regions.

FFAs detected in the whole brain have been explained according to their specific region and relative concentrations. Prospective molecular formulas of potential FFAs were generated based on accurate *m*/*z* using the Thermo Xcalibur 2.1 Software. Annotation was performed as follows: the information (RI, accurate *m*/*z*) of detected paired peaks was compared to in-house chemically labeled standards library (http://59.110.238.58/search.php?tdsourcetag=s_pctim_aiomsg). A total of 56 peak pairs were matched with library data (No. 1–56 in Table S1) of which 38 of FFAs were found to be present in every region (highlighted in the Table S1). There were 18 saturated fatty acids, six monounsaturated fatty acids, 10 polyunsaturated fatty acids (PUFAs), and four bile acids. A total of 10 PUFAs were found to be present in every region, since they play an important role in the brain. Bile acids may be transferred to the brain through the blood or hepatic gut brain axis, which is consistent with a previous study [29,30]. Further research is needed to examine the potential function of bile acids in the brain. Remaining peak pairs were assigned by searching the molecular formulas in the METLIN database (https://metlin.scripps.edu/landing page.php) and 339 peak pairs matched to one or more chemical structures (No. 57–395 in Table S1). Formulae for 613 out of 1008 detected peak pairs were not found in the METLIN database. This suggests that there may be some new fatty acids existing in the brain that require further investigation (No. 396–1008 in Table S1).

### 2.2. Common FFAs between Two Regions

**Figure 3:**
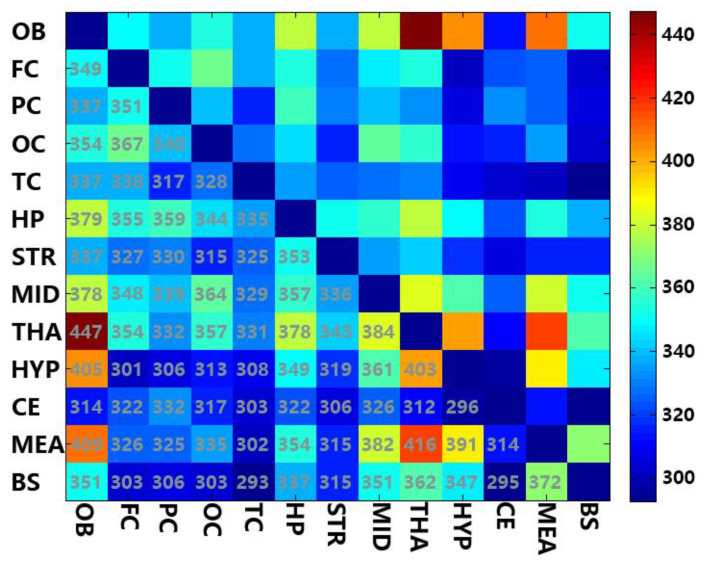
A heat map of common fatty acid species between two different brain regions.

The species of FFAs were compared between two different brain regions with each pair (Figure 3). There were similar fatty acids between two different brain regions, which are common FFAs. THA and OB have 447 common potential fatty acids (the most common pair). The total number of fatty acids present commonly in HYP and OB, THA and HYP, and MEA and THA were found to be more than 400. The regions with less than 300 common fatty acids were found to be BS and TC, HYP and CE, and BS and CE. The common fatty acids of OB vs. THA, HYP, and MEA are more than in the other regions. That may relate to the function that the two regions participate in: THA mediates visceral-somatic reflex activity, which is associated with the sense of smell and part of it is connected to OB whereas; HYP is related to feeding which depends on the sense of smell in rats [31,32,33]. The mutual function of two different brain regions may lead to similar composition. That might be the reason they have more common fatty acids.

**Figure 4:**
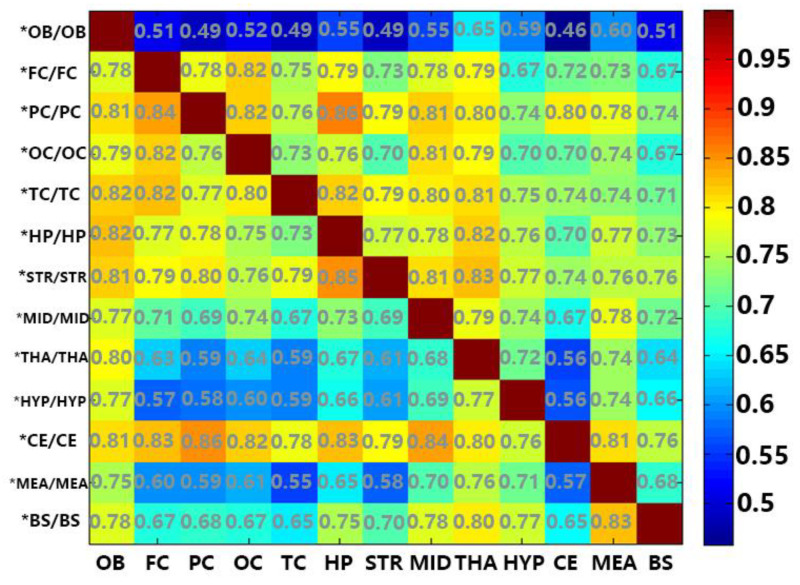
A heat map of the ratio between common fatty acid species among crossed regions and the total species in the row related brain region. "*”: the common fatty acid species between the intersection of the brain region on abscissa axis and the one on vertical axis; “/“: the proportion of the common fatty acid species and the region after ‘/’.

However, we cannot deny the fact that these regions have more fatty acids within them, which makes it easier to match more fatty acids with the other regions. Thus, the ratio of the number of A region divided by common FFAs amounting to between A and B is more likely to show a similarity to A region and B region. Figure 4 shows the percentage of common fatty acids between two different regions divided by the vertical shaft region’s potential fatty acids. For instance, 0.78 from the first column and second row in Figure 4 means the common FFAs between FC and OB are 78% of the FFAs in FC. The common FFAs are 51% of the FFAs in OB (first row and second column in Figure 4). We demonstrated that the OB and most of the other regions have a higher proportion of common fatty acids vs. the region being compared, while they have a lower proportion vs. the OB. Nearly all regions and CE have a higher proportion of common fatty acids vs. CE. This may be due to the fact that the OB has the most abundant free fatty acids compared to the other regions, while CE has the least. This factor can also explain why common free fatty acids of OB vs. THA, HYP, and MEA are apparently more than others.

### 2.3. Relative Quantitation of FFAs in OB, OC, HP, and CE

Sense of smell is one of the earliest senses present in organisms and plays an important role in rats for living, breeding, and defensive activities. These activities are the bases of a rat’s life and involve the localization of the visual system, the participation in learning and memory as well as the movement system. The OB is a part of smelling system, OC is the visual cortex, HP is a key part of learning and memory, and CE is a motor control center; these were selected as the four representative brain regions to quantitatively analyze the various contents of FFAs. One HP and two CE samples were removed while processing the data for exploratory analysis, since the standards added (C5:0–C24:0) were detected partially in these three samples. The normalized contents of FFAs were analyzed by PCA, OPLS-DA, and one-way ANOVA.

**Figure 5:**
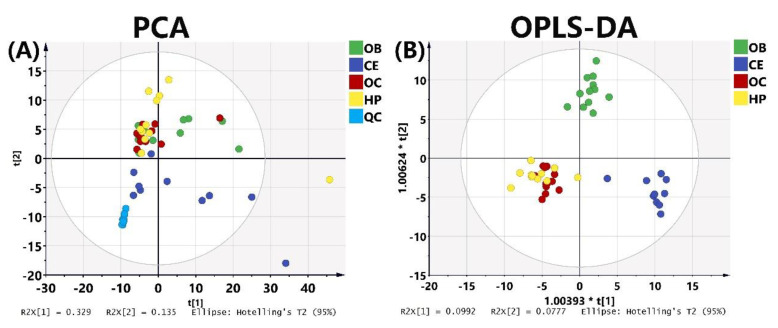
(**A**) Principal component analysis (PCA0 score plot of 4 brain regions (R^2^X = 0.749, Q^2^ = 0.541). (**B**) Orthogonal partial least squares–discriminant analysis OPLS-DA score plot of 4 brain regions (R^2^X = 0.698, R^2^Y = 0.88, Q^2^ = 0.71). Red dots represent OC; green dots represent OB; yellow dots represent HP; dark blue dots represent CE; light blue dots represent QC.

**Figure 6:**
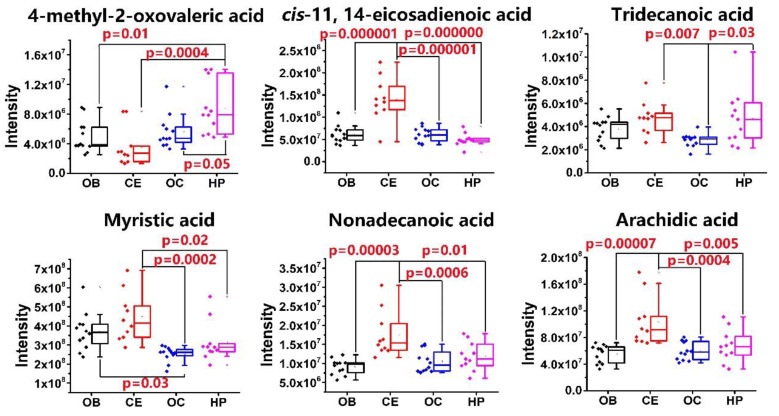
Relative concentration alternation of free fatty acids in 20 mg of rat. Boxplots and scatter plots of six significantly altered FFAs. *p* values were calculated by one-way ANOVA.

Results of PCA (Figure 5A) reveal that the QC sample (Figure 5A, light blue dot) united with the analytical samples, indicating that the instrument was stable during the whole analysis and thus ensured the reliability of the results. CE (dark blue dot) is clearly distinguished on the basis of FFAs content, while the other three brain regions could not be clearly distinguished (Figure 5A). OPLS-DA analysis (Figure 5B) shows that the OB and CE are distinguished clearly on the basis of the fatty acid contents. According to the VIP value > 1.0 in OPLS-DA analysis and *p* value < 0.05 in one-way ANOVA analysis, six fatty acids, including four saturated fatty acids ((C13:0, C14:0, C19:0, C20:0), *cis*-11,14-eicosadienoic acid and 4-Methyl-2-oxovaleric acid) were statistically significant different in four brain regions (Figure 6).

Eicosadienoic acid (Δ11,14-20:2; EDA) is a naturally occurring *n*-6 polyunsaturated fatty acid (PUFA) and can modulate the metabolism of PUFA and change the responsiveness of macrophages to an inflammatory stimulation [34]. EDA was found to be different between moderating Alzheimer’s disease (AD) or dementia with Lewy bodies and cognitively normal age-matched controls [35]. Ketoleucine, also known as 4-Methyl-2-oxovaleric acid, arises from the incomplete breakdown of branched-chain amino acids and acts both as a neurotoxin and a metabotoxin. Maple syrup urine disease (MSUD) is associated with chronically high levels of ketoleucine. Without treatment, it leads to severe brain damage in infancy and abnormal behaviors and moods in older individuals [36]. Ketoleucine was reported to be higher in the cerebrospinal fluid from Amyotrophic lateral sclerosis or Parkinson’s disease patients [37]. The content difference of free fatty acids may relate to the difference in regional activity.

## 3. Material and Method

### 3.1. Study Design

**Figure 7:**
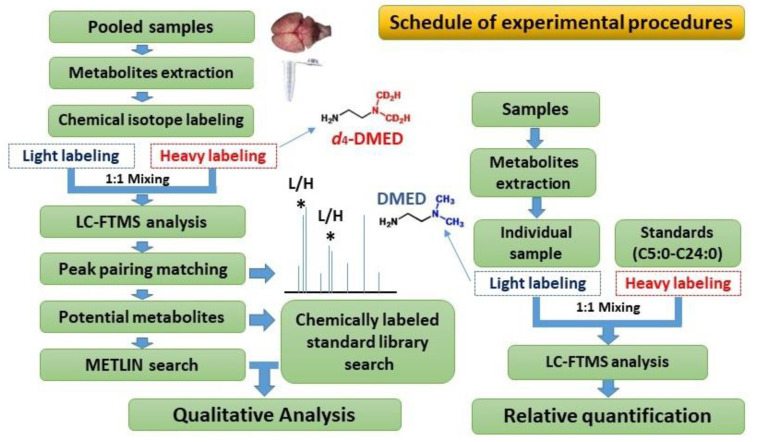
Workflow for determination of fatty acids in rat brain by the chemical isotope labeling liquid chromatography–mass spectrometry (CIL-LC-MS) method; 2-dimethylaminoethylamine (DMED) and *d*_4_-2-dimethylaminoethylamine (*d*_4_-DMED) were utilized to facile label free fatty acids. L, DMED labeled peaks; H, *d*_4_-DMED labeled peaks; “*” means a pair of peaks that will be extracted as a potential fatty acid.

A total of 12 different cerebral regions and medulla of rats were selected as study materials. The CIL-LC-MS technique was employed for qualitative and quantitative investigation of the variations in types and contents of FFAs in different brain regions. A pair of isotopic probes (2-dimethylaminoethylamine (DMED) and *d_4_*-2-dimethylaminoethylamine (*d*_4_-DMED)) containing carboxylic acid reactive group, were used to label free fatty acids, based on amidation. The detailed workflow of the current work is shown in Figure 7.

### 3.2. Chemicals and Reagents

All fatty acid standards were purchased from Sigma (St. Louis, MO, USA) and J&K Chemical (Beijing, China). Analytical grade formic acid (FA), ethyl acetate (EA), triethylamine (TEA), 2-chloro-1-methylpyridinium iodide (CMPI), and DMED were obtained from Sinopharm Chemical Reagent Co., Ltd. (Shanghai, China). Isotope labeling reagent of *d*_4_-DMED was synthesized according to our previously reported method [27]. HPLC-grade acetonitrile (ACN) and methanol were purchased from TEDIA Co., Inc (Fairfield, OH, USA). Water used for analysis was purified by a Milli-Q apparatus (Millipore, Milford, CT, USA). Stock solutions of TEA (20 mmol/L), CMPI (20 mmol/L), DMED (40 mmol/L), and *d*_4_-DMED (40 mmol/L) were prepared in HPLC-grade ACN. Stock solutions of standard organic acids were prepared in HPLC-grade ACN with a concentration of 1.0 mg/mL for each and stored at −20 °C until analyzed.

### 3.3. Brain Sample Collection and Pre-Treatment

The experiments were approved by the Animal Ethics Committee at the Wuhan Institute of Physics and Mathematics and met all the prescribed guidelines. Fifteen Sprague Dawley male rats (3 for the qualitative study and 12 for the quantitative study) were purchased from the Center for Disease Control of Hubei Province and adapted to the environment for a week in an animal facility with regulated temperature, humidity, and light cycle and with free access to water and food. The age of rats was around seven weeks old and the weight between 200 and 220 g at the start of the experiment.

On the experimental day, three animals were initially anesthetized with overdose of isoflurane, and the level of anesthesia was verified with a pinch applied to the hind leg. After that, three anesthetized animals were euthanized with the head-focused microwave method using a commercially available microwave machine (1 kW, Tangshan Nanosource Microwave Thermal Instrument Manufacturing Co. Ltd., Hebei, China). The microwave method was used in this study to avoid post-mortem release of fatty acids [9], since Farias reported that AA increased 18-fold after brain ischemia [38]. Post-mortem release of fatty acids may lead to a substantial increase in concentration of some FFAs. This can result in further increase in peaks of some fatty acids, which may suppress the other nearby fatty acid peaks. After cooling down the rat head to room temperature with dry ice, the whole brain was collected and manually dissected into 13 different brain regions. The dissected brain regions were: olfactory bulb (OB), frontal lobe (FC), parietal lobe (PC), occipital lobe (OC), temporal lobe (TC), hippocampal (HP), thalamus (THA), hypothalamic (HYP), midbrain (MID), striatum (STR), cerebellum (CE), medulla (MEA), and brainstem (BS). All samples were weighed and stored at −80 °C until further analysis. In order to decrease the variability among the different animals, brain samples from the same region of different animals were pooled together and transferred to a 5 mL centrifuge tube. Then, 1mL pre-cooled saline solution was added and the mixture grinded in an ice bath. After that, 2 mL of EA was further added, and the mixture vortexed for 3 min for the extraction of fatty acids. The resulting mixture was centrifuged at 13,000 *g* for 3 min at 4 °C, and the supernatant was collected. The extraction procedure with EA was repeated twice, and the supernatants were combined together and vortexed again. The supernatants were transferred to a new centrifuge tube according to the brain weight ensuring that the transferred liquid was extracted from 100 mg of tissue. The transferred supernatants were dried under nitrogen gas and residues were used for subsequent chemical labeling.

Twelve rats were anesthetized with an overdose of isoflurane and then decapitated. The Microwave method was not applied here in order to avoid any possible chemical changes in free fatty acids. The brain was separated, collected quickly, and put above the dry ice in order to be dissected into different regions. The samples were stored at −80 °C for further analysis after flash-freezing with liquid nitrogen. The four representative cerebral regions OB, HP, OC, and CE were grinded on dry ice independently, centrifuged, and the supernatants collected by following the same procedure as in the previous step. The collected supernatant extracted from 20 mg of each sample was then dried under nitrogen gas. A quality control (QC) sample was prepared by equally mixing brain samples from four different regions.

### 3.4. Labeling of Free Fatty Acids with DMED and d4-DMED

For qualitative analysis, the labeling of DMED and *d*_4_-DMED was performed by following a previously described method [27,28]. The specific labeling process was as follows: 200 μL ACN was added to brain residues and the mixtures vortexed for 3 min. The mixture was divided into two equal parts. A 20 μL of TEA (20 mmol/L) and 10 μL CMPI (20 mmol/L) were added to the solution then vortexed and incubated at 40 °C for 5 min. Then 20 μL of DMED (40 mmol/L) or 20 μL *d*_4_-DMED (40 mmol/L) was added, and the solution was incubated at 40 °C for 1 h. The resulting solution was dried under nitrogen and re-dissolved in 100 μL ACN/water (*v*/*v*, 1/9) and left for LC-Orbitrap MS analysis.

For relative quantitative analysis, samples were labelled with DMED by following the above described procedure. To overcome the error caused by retention time drifts in LC analysis, retention indices were adopted for correction by adding straight-chain n-alkanoic acids (C5:0-C24:0) labeled by *d*_4_-DMED.

### 3.5. UPLC-MS Analysis

Brain samples were analyzed using the Ultra Performance Liquid Chromatography (UPLC)-LTQ-Orbitrap MS system consisting of a LTQ-Orbitrap Elite mass spectrometer (Thermo Scientific, Waltham, MA, USA) with an electrospray ionization source and a Dionex Ultimate 3000 UHPLC system (Thermo Scientific, Sunnyvale, CA, USA). Data acquisition and processing were performed with Thermo Xcalibur Software (version 2.1, Thermo Fisher Scientific, Inc., Waltham, MA, USA). An Acquity UPLC BEH C18 Column (2.1 × 50 mm, 1.7 μm, Waters, Milford, CT, USA) was used for HPLC separation with a flow rate of 0.4 mL/min at 40 °C. FA in water (0.1%, *v*/*v*, solvent A), and ACN (solvent B) was used as mobile phases. A gradient of 0–5 min at 5% B, 5–48 min from 5 to 95% B, 48–53 min at 95% B, 53–55 min from 95 to 5% B, and 55–60 min at 5% B was applied. The injection volume was 10 μL. The labeled samples were detected in positive ion full scan mode in the range of *m*/*z* 180–650 at resolution of 60,000. ESI conditions employed were as follows: capillary temperature, 350 °C; spray voltage, 3.5 kV; sheath gas flow, 35 arbitrary; auxiliary gas flow, 15 arbitrary; heater temperature, 300 °C.

### 3.6. Data Analysis

Thermo sieve 2.1 Software (Thermo Fisher Scientific, Waltham, MA, USA) was used to extract MS spectral peaks of raw data to generate the information about detected fatty acid compounds with accurate m/z, peak intensity, and retention time (RT). RTs were calibrated by retention indices (RIs) as reported [28] to overcome the drifting of retention times. Peak pairs extraction was operated with 4.025 Da mass difference, similar peak intensities and RIs by using an in-house MATLAB-based software. Prospective molecular formulas of DMED-labeled fatty acids were generated based on the accurate m/z using the Thermo Xcalibur 2.1 Software. Mass tolerance of 5.0 mDa was set, and the elements C, H, N, and O were used. In-house chemically labeled standards library (http://59.110.238.58/search.php?tdsourcetag=s_pctim_aiomsg) was employed for fatty acids identification from brain samples.

Principal component analysis (PCA) and orthogonal partial least squares–discriminant analysis (OPLS-DA) were used for the exploratory analysis of the data of relative concentrations by using SIMCA 14.1 Software (Umetrics AB, Umea, Sweden). IBM SPSS statistics 19.0 was performed for one-way ANOVA analysis. Variable importance for projection (VIP) value of OPLS-DA model more than 1.0 and *p*-value obtained by one-way ANOVA less than 0.05 were considered as statistically significant difference of fatty acids. Parts data were dealt with using data transformations (square-root or base-10 logs) to make variables fit a normal distribution and homoscedascity test before one-way ANOVA.

## 4. Conclusions

The CIL-LC-MS technique was applied to analyze free fatty acids in 12 brain regions and the medulla. At the end, a total of 1008 potential free fatty acids were found in the whole brain, and of those, 56 fatty acids were positively identified. Thirty-eight out of 56 were found to be common free fatty acids present in every brain region. Among different brain regions, the olfactory bulb contains the most abundant fatty acids, while the cerebellum has the least. Different cortex parts have similar amounts but different species of fatty acids. In addition, we quantitatively explored the distribution of fatty acids in the OB, OC, HP, and CE. Six fatty acids were found to be significantly different. The compositions of the fatty acids varied among different regions. This study may provide a more comprehensive understanding of the distribution of fatty acids in brain regions and thus provide important information for neurochemical studies.

## Supplementary Materials

The following are available online, Table S1: Content of the detected potential fatty acids in brain.
